# Attachment of class B CpG ODN onto DOTAP/DC-chol liposome in nasal vaccine formulations augments antigen-specific immune responses in mice

**DOI:** 10.1186/s13104-017-2380-8

**Published:** 2017-01-26

**Authors:** Rui Tada, Shoko Muto, Tomoko Iwata, Akira Hidaka, Hiroshi Kiyono, Jun Kunisawa, Yukihiko Aramaki

**Affiliations:** 1 Department of Drug Delivery and Molecular Biopharmaceutics, School of Pharmacy, Tokyo University of Pharmacy and Life Sciences, 1432-1, Horinouchi, Hachioji, Tokyo 192-0392 Japan; 2 Division of Mucosal Immunology and International Research and Development Center for Mucosal Vaccines, Department of Microbiology and Immunology, The Institute of Medical Science, The University of Tokyo, Tokyo, Japan; 3 Laboratory of Vaccine Materials, National Institutes of Biomedical Innovation, Health and Nutrition (NIBIOHN), Osaka, Japan

**Keywords:** Mucosal adjuvant, Cationic liposome, CpG ODN, Intranasal immunization, Vaccine

## Abstract

**Background:**

To overcome infectious diseases, the development of mucosal vaccines would be an effective strategy, since mucosal surfaces are the entry site for most pathogens. In general, protein antigens show inherently poor immunogenicity when administered by the mucosal route. Therefore, co-administration of an appropriate mucosal adjuvant is required to exert immune responses toward pathogen-derived antigens effectively. However, the development of a safe and effective mucosal adjuvant system is still challenging. Although, recent studies reported that oligodeoxynucleotides (ODNs) containing immunostimulatory CpG motifs (CpG ODNs) act as potent mucosal adjuvants and are useful in the formulation of nasal vaccines, there are some disadvantages. For instance, the administration of phosphorothioate (PS)-modified CpG ODNs can induce adverse systemic effects, such as splenomegaly, in a dose-dependent manner. Therefore, a reduced dose of CpG ODN might be crucial when used as vaccine adjuvant for clinical purposes. Therefore, we prepared a CpG ODN-loaded cationic liposome, and evaluated its mucosal adjuvant activity.

**Results:**

We prepared a CpG ODN-loaded DOTAP/DC-chol liposome that was stable during our experiments, by mixing CpG ODNs and liposomes at an N/P ratio of 4. Further, we demonstrated that the attachment of class B CpG ODN to the DOTAP/DC-chol liposomes synergistically enhanced antigen-specific IgA production in the nasal area than that induced by CpG ODN and DOTAP/DC-chol liposomes alone. The endpoint titers were more than tenfolds higher than that induced by either single CpG ODN or single DOTAP/DC-chol liposomes. Additionally, although serum IgG1 responses (indicated as a Th2 response) remained unchanged for DOTAP/DC-chol liposomes and CpG ODN-loaded DOTAP/DC-chol liposomes, the CpG ODN-loaded DOTAP/DC-chol liposomes synergistically induced the production of serum IgG2a (indicated as a Th1 response) than that by the individual liposomes.

**Conclusions:**

We conclude that the advantage of using DOTAP/DC-chol liposome harboring CpG ODN is it induces both antigen-specific mucosal IgA responses and balanced Th1/Th2 responses. Therefore, such a combination enables us to resolve the adverse effects of using CpG ODNs (as a mucosal adjuvant) by reducing the overall dose of CpG ODNs. Further, the biodegradable and essentially non-antigenic nature of the liposomes makes it superior than the other existing mucosal adjuvants.

## Background

Infections are one of the most significant risk factors for human lives and the second leading cause of death worldwide [1, 2]. Much focus has been on intranasal mucosal vaccines due to their capacity to elicit immune responses at the site pathogen entry and systemic infection [3–9]. Therefore, mucosal vaccines would be an effective strategy to fight infectious diseases caused by pathogenic microbes invading through mucosal tissues of host including fungi, bacteria, and viruses. However, when administrated by the mucosal route, protein antigens show inherently poor immunogenicity [10]. Therefore, co-administration of an appropriate mucosal adjuvant with protein antigens is needed to exert immune responses to the antigens effectively [8]. Yet, adjuvants used experimentally for a long time are mainly pathogen-derived toxins, such as the cholera toxin and heat-labile enterotoxin, evokes unexpected side effects caused by their toxicity and antigenicity [11, 12]. In this context, the development of safe and effective mucosal adjuvants is fundamental to prevent infections by the use of mucosal vaccines in the clinic.

Recent studies reported that intranasal administration of synthetic oligodeoxynucleotides (ODNs) containing immunostimulatory CpG motifs (CpG ODNs) act as a mucosal adjuvant via the recognition by toll-like receptor 9 (TLR9) in mice. CpG ODN are classified into 5 classes based on their specific sequences and effects, namely class A, B, C, P, and S. Class B CpG ODN exhibits strong B cell activation resulting in enhanced antibody production [11, 13–20]. However, there are some disadvantages with the use of CpG ODN as adjuvants as it is quickly eliminated by host nucleases present in tissues and blood under physiological conditions. To prevent CpG ODN degradation in the host body, the phosphodiester (PO) backbone within CpG ODNs is chemically modified to the phosphorothioate (PS) linkage, reducing the sensitivity of CpG ODN to nuclease digestion, and thus increasing its adjuvant activities [21–24]. Despite the chemical modification to improve CpG ODN stability in the host, free CpG ODN still has the following disadvantages: (1) a reduced affinity to TLR9 for PS-modified CpG ODN compared to original CpG ODN [25], (2) The administration of CpG ODN in mice resulted in splenomegaly in a sequence-specific and dose-dependent manner [26], (3) PS–modified CpG ODNs are still susceptible to nuclease [21], (4) PS-modified CpG ODN transiently activates the host complement system, that could lead to cardiovascular collapse and death [27, 28]. Consequently, a reduced dose of PO-modified CpG ODNs with stable form might be required for the usage as a safe and efficient mucosal adjuvant.

We have recently reported that intranasal administration of an antigen with the cationic liposomes composed of 1,2-dioleoyl-3-trimethylammonium-propane (DOTAP) and 3β-[*N*-(*N*′,*N*′-dimethylaminoethane)-carbamoyl] (DC-chol) induced antigen-specific mucosal and systemic antibody responses in mice. We further revealed that this cationic liposome possesses Th2-type adjuvant activities. However, the effects of nasal IgA production and Th1-type of immune responses are relatively weak compared with those of the cholera toxin, a well-known potent experimental mucosal adjuvant [29]. We hypothesized that the combination of CpG ODN and the cationic liposomes would exert a more efficient IgA response in the nasal area and a Th1-type immune response in the systemic system.

To develop a potent mucosal adjuvant system with cationic liposomes, we prepared a class B CpG ODN-loaded cationic liposome not only to enhance IgA production in mucosal tissues and induce Th1-type immune response due to IgG2a production, but also to reduce the overall dose of CpG ODNs without any loss of mucosal adjuvant properties. Subsequently, we evaluated the activities of the mucosal adjuvant when administered intranasally with ovalbumin as a model antigen, and examined the induction of OVA-specific antibody production in both mucosal and systemic in mice.

## Methods

### Animals and materials

Female BALB/c mice (6-weeks old) were purchased from Japan SLC (Shizuoka, Japan). The mice were housed in a specific pathogen-free environment. All animal experiments followed the guidelines for laboratory animal experiments of the Tokyo University of Pharmacy and Life Sciences, and each experimental protocol was approved by the Committee for Laboratory Animal Experiments at the institution (P14–32 and P15–33). DOTAP and DC-chol were purchased from Avanti Polar Lipids (Alabama, USA). Class B CpG ODN 1826 (5′-tccatgacgttcctgacgtt-3′; small letters indicate phosphorothioate linkage, underlining indicates the CpG motif) was synthesized by the Hokkaido System Science Co., Ltd. (Hokkaido, Japan). Ovalbumin from egg white (OVA) was obtained from Sigma-Aldrich (St. Louis, USA).

### Preparation of the cationic liposomes

Liposomes were prepared as described previously [29, 30]. Briefly, 10 μmol of total lipids (DOTAP:DC-chol at 1:1 mol ratios) were evaporated to dryness in a glass tube and desiccated for at least 1 h in vacuo. The obtained lipid films were hydrated by the addition of 250 μl of phosphate-buffered saline (PBS) and then vortexed at room temperature (RT) for 5 min. The multilamellar vesicles were extruded 10 times passing through a 100 nm-pore polycarbonate membrane (ADVANTEC, Tokyo, Japan) and sterilized by 0.45 μm of filter membranes (IWAKI, Tokyo, Japan). The particle size and ζ-potential of the liposomes prepared in this study were measured by NICOMP 380 ZLS (Particle Sizing Systems; Port Richey, USA).

### Preparation of CpG ODN-loaded DOTAP/DC-chol liposomes

One microgram of CpG ODN solution was added into the prepared DOTAP/DC-chol liposomes described above (10 μl of PBS), and incubated for 5 min at RT. After incubation, the samples were ready to use for the measurement of their particle size and ζ-potential stated above, and assessment of their mucosal adjuvant activities described below.

### Agarose gel retardation assay

CpG ODN-loaded DOTAP/DC-chol liposomes were prepared by combining CpG ODN and DOTAP/DC-chol liposomes for 5 min at RT. The complex formation was determined by an agarose gel retardation assay. Complexes and free CpG ODN at various N/P ratio were applied onto 1% agarose gels. The agarose gels were stained and visualized with the GelRed nucleic acid gel stain (Wako Pure Chemical Industries).

### Immunization and sampling schedule

Mice were divided into five groups and nasally immunized as follows: (1) PBS, (2) OVA alone, (3) OVA plus DOTAP/DC-chol liposomes, (4) OVA plus CpG ODN, or (5) OVA plus CpG-liposomes (N/P ratio = 4). Each group of mice was immunized once a week. To obtain serum samples, blood samples were collected. The blood was allowed to clot at 25 °C for 30 min followed by 4 °C for 60 min and then the serum was separated by centrifugation at 1200*g* for 30 min and stored at −80 °C until ELISA assays. Nasal wash fluid samples were collected using 200 µl of PBS solution as described earlier [29, 31, 32].

### ELISA for detecting OVA-specific antibody production [29]

A 96-well Nunc MaxiSorp plate (Thermo Scientific, MA, USA) was coated with 1.25 μg of OVA in 0.1 M carbonate buffer (pH 9.5) and incubated overnight at 4 °C. The plate was then washed with PBS containing 0.05% Tween 20 (PBST) and blocked with 1% bovine serum albumin (BSA; Wako Pure Chemical Industries) containing PBST (BPBST) at 37 °C for 60 min. The plate was washed and incubated with serum samples for 60 min at 37 °C. It was then washed with PBST, treated with peroxidase-conjugated anti-mouse IgA, IgG, IgG1, or IgG2a secondary antibody (SouthernBiotech, Alabama, USA) in BPBST, and developed using a tetramethylbenzidine (TMB) substrate system (KPL, Maryland, USA). Color development was terminated using 1 N phosphoric acid, and the optical density was measured at 450 nm with 650 nm of reference.

### Statistical analysis

Statistical differences were assessed by Kruskal–Wallis with Dunn’s post hoc test. *p* values less than 0.05 were considered significant.

## Results and discussion

### Characterization of CpG ODN-loaded DOTAP/DC-chol liposomes

**Fig. 1:**
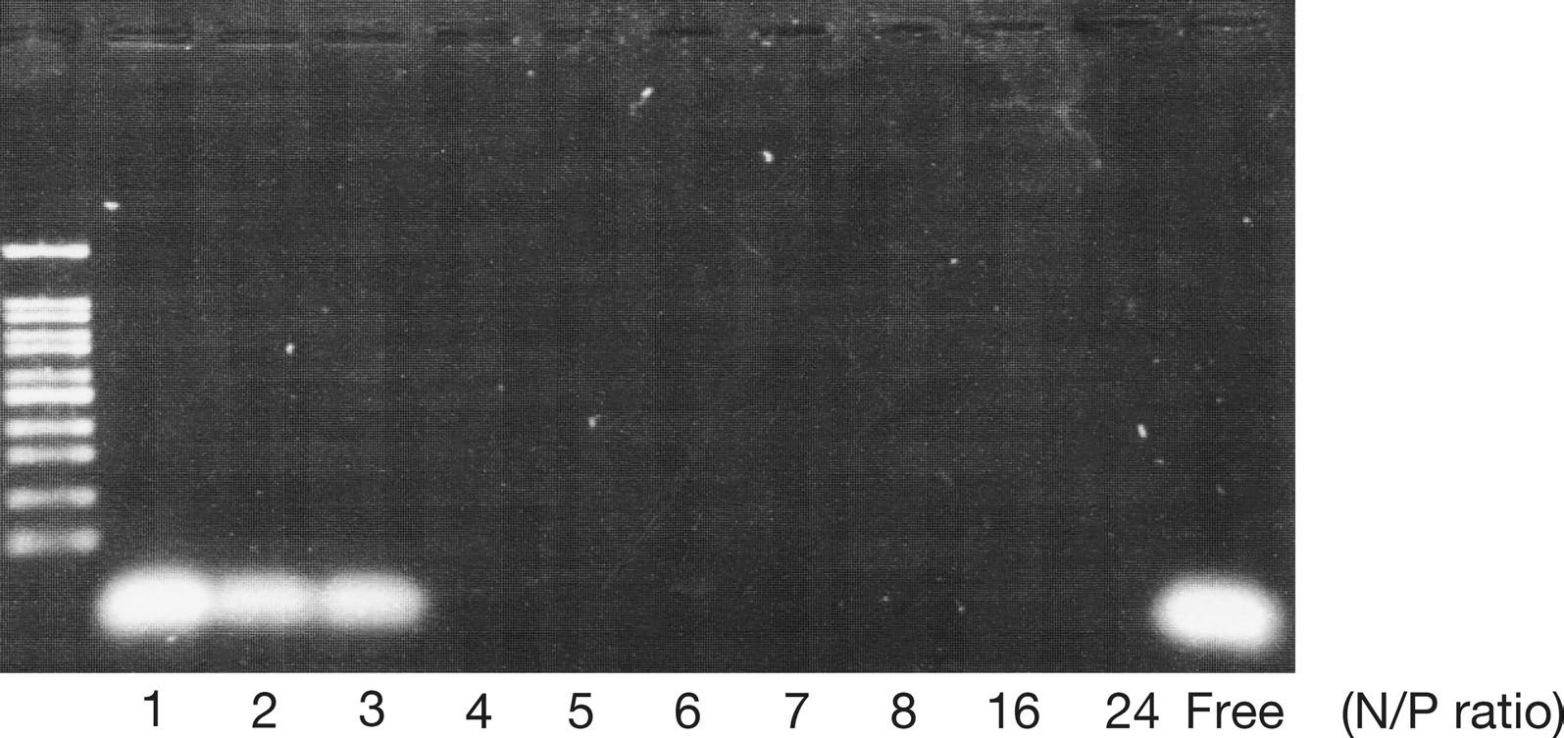
Agarose gel retardation assay of the prepared CpG ODN-loaded DOTAP/DC-chol liposomes with various N/P ratios. The prepared CpG ODN-loaded DOTAP/DC-chol liposomes were loaded onto a 2% agarose gel and then ODN bands were visualized by staining with GelRed Nucleic Acid Gel Stain

**Fig. 2:**
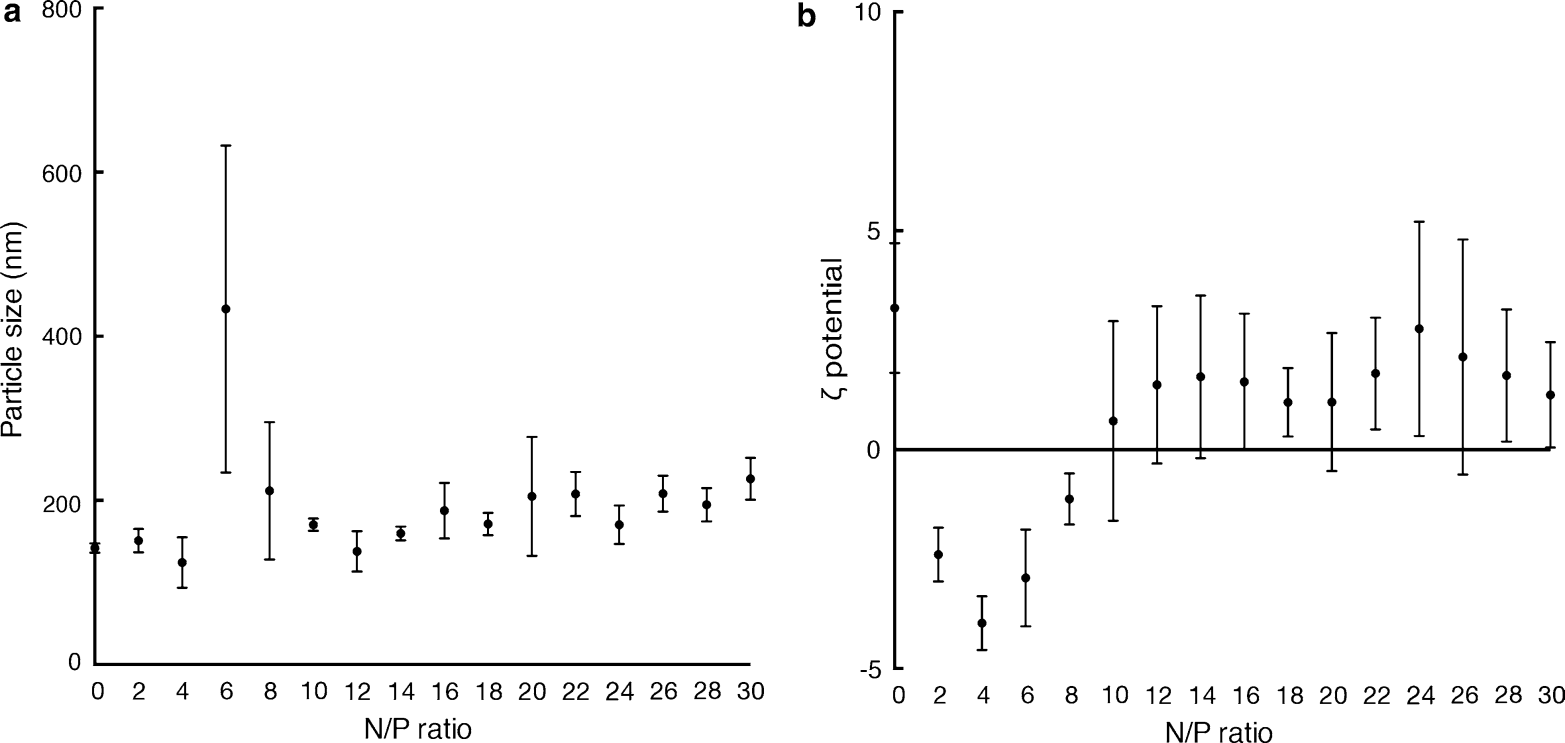
Distribution of particle size (**a**) and ζ-potential (**b**) of the prepared CpG ODN-loaded DOTAP/DC-chol liposomes with various N/P ratios. The particle size and ζ-potential of the prepared CpG ODN-loaded DOTAP/DC-chol liposomes were measured by NICOMP 380 ZLS. The particle size and ζ-potential are expressed as the mean ± standard error for samples assayed in triplicate

First, loading capacity of DOTAP/DC-chol liposome with class B CpG ODN in PBS solution was evaluated by an agarose gel retardation assay. The liposome/CpG ODN ratio was expressed as the N/P ratio that was dependent on the ratio of nitrogen (N) in the liposomal lipids to the phosphates (P) in the nucleic acids of CpG ODNs. At the N/P ratio of 3, we still observed free CpG ODN that was not complexed with the liposomes. Then at the N/P ratio of 4, free CpG ODN could not be detected, indicating all CpG ODN was loaded onto the liposomes in this N/P ratio (Fig. 1). In case of higher N/P ratios, DNA loaded onto nanoparticles can be easily detected on the agarose gel owing to retention of the complex within the wells. However, the retained DNA cannot be visualized in the gel image shown in Fig. 1 because DNA complexed with nanoparticles diffuses into the staining buffer during the staining process, thus hampering visualization. In addition to the agarose gel retardation assay, particle size and ζ-potential analyses of the CpG ODN-loaded DOTAP/DC-chol liposomes at various N/P ratios were conducted (Fig. 2). The DOTAP/DC-chol liposomes alone in PBS solution were positively charged (3.6 ± 2.3 mV). Increasing quantities of CpG ODN led to a reduction in this positive charge of DOTAP/DC-chol liposomes until an N/P ratio of 4. Subsequently, continuous increase in the N/P ratio (increase in the liposome quantity) leads to rise in the average ζ-potential as we tested in the present study (up to N/P ratio of 24). Collectively, the N/P ratio of 4 seemed that CpG ODN at the surface of DOTAP/DC-chol liposomes was saturated. Additionally, the CpG ODN-loaded DOTAP/DC-chol liposomes were stable at an N/P ratio of 4 during course of experiments, however with an increased N/P ratio they became unstable in terms of their particle size, possibly due to their positively charged ζ-potential (data not shown). Therefore, we hereafter examined mucosal adjuvant effects of CpG ODN-loaded DOTAP/DC-chol liposomes at the N/P ratio of 4. At this N/P ratio, CpG ODN-loaded DOTAP/DC-chol liposomes had a particle size of 124.2 ± 53.3 nm with a ζ-potential of −3.9 ± 4.7 mV (Table 1). It is noteworthy that the distribution of number-based particle size of CpG ODN-loaded DOTAP/DC-chol liposomes at an N/P ratio of 4 showed almost narrow distribution. In contrast, distribution of particle size appeared broader when the N/P ratio was increased, indicating that the nanoparticles were aggregated (data not shown). These sizes, at the N/P ratio of 4, would be suitable, since a possible mechanism underlying the advantage of CpG ODN attached to a nanoparticle-like liposome could be an efficient delivery to antigen-presenting cells (APCs) such as dendritic and B cells [33, 34]. APCs are able to take up the nano-sized particles efficiently [35]. We have already reported that DOTAP/DC-chol liposomes are preferentially uptaken by DCs in vivo [29].

**Table 1:** Physicochemical properties of the CpG ODN-loaded DOTAP/DC-chol liposomes used in this study

	N/P ratio	Particle size (nm)	ζ-potential (mV)
CpG ODN-loaded DOTAP/DC-chol liposome	0	137.9 ± 11.6	4.0 ± 2.1
DOTAP/DC-chol liposome	4	124.2 ± 53.3	−3.9 ± 4.7

### Antigen-specific nasal IgA and serum IgG responses after administration of OVA with class B CpG ODN

**Fig. 3:**
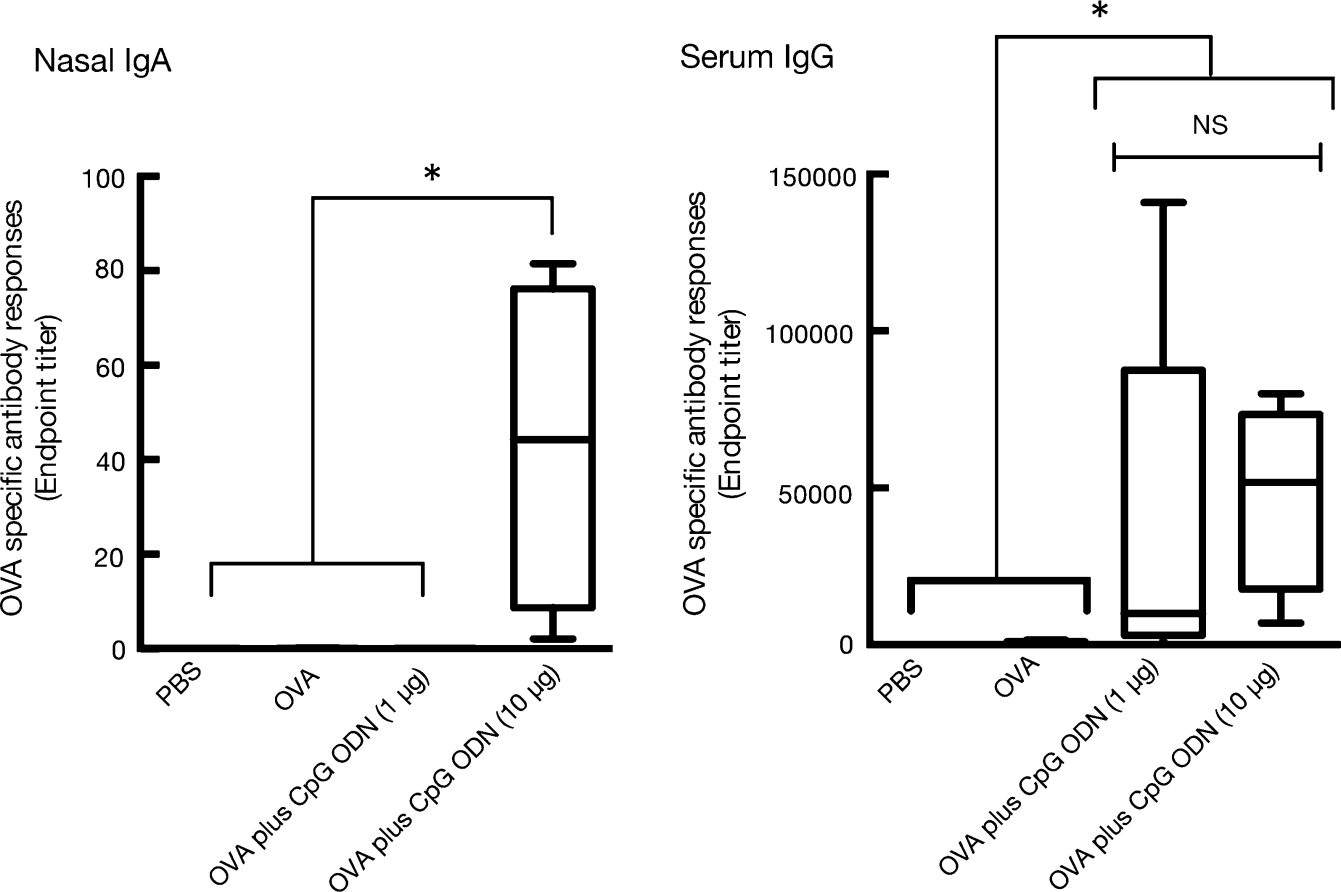
Mucosal adjuvant activity of CpG ODN assessed by the induction of OVA-specific nasal IgA and serum IgG in BALB/c mice. BALB/c female mice were immunized intranasally with OVA (5 µg/mouse) alone, or OVA (5 µg/mouse) plus CpG ODN (1 or 10 µg/mouse) on days 0 and 7. Serum and nasal washes were collected on day 14. The anti-OVA IgA level in nasal washes and anti-OVA IgG, IgG1, and IgG2a levels in serum were detected by ELISA assay as described in the “Methods” section. The data were obtained from three independent experiments. The *box-plot* shows the median value with the 25th–75th percentiles and the *error bars* indicate the 5th–95th percentiles. *Symbols in the box plots* represent individual mice (PBS, n = 8; OVA, n = 8; OVA plus CpG ODN (1 µg/mouse), n = 8; OVA plus CpG ODN (10 µg/mouse), n = 4). Significance was assessed using the Kruskal–Wallis with Dunn’s post-hoc test: *p < 0.05, *NS* not significant

We next investigated a required dose of class B CpG ODN to exhibit the mucosal adjuvant effects when intranasally administrated with OVA in BALB/c mice. As shown in Fig. 3, intranasal treatment of 10 µg of CpG ODN exerted the production of nasal IgA (endpoint titer with median value; 44.3); however, intranasal administration of 1 µg of CpG ODN did not induce any nasal IgA production. Meanwhile, serum IgG levels of mice that received all dose ranges of CpG ODN examined in this study did not show any differences.

### Induction of antigen-specific nasal IgA and serum IgG after administration of OVA with CpG ODN-loaded DOTAP/DC-chol liposomes

**Fig. 4:**
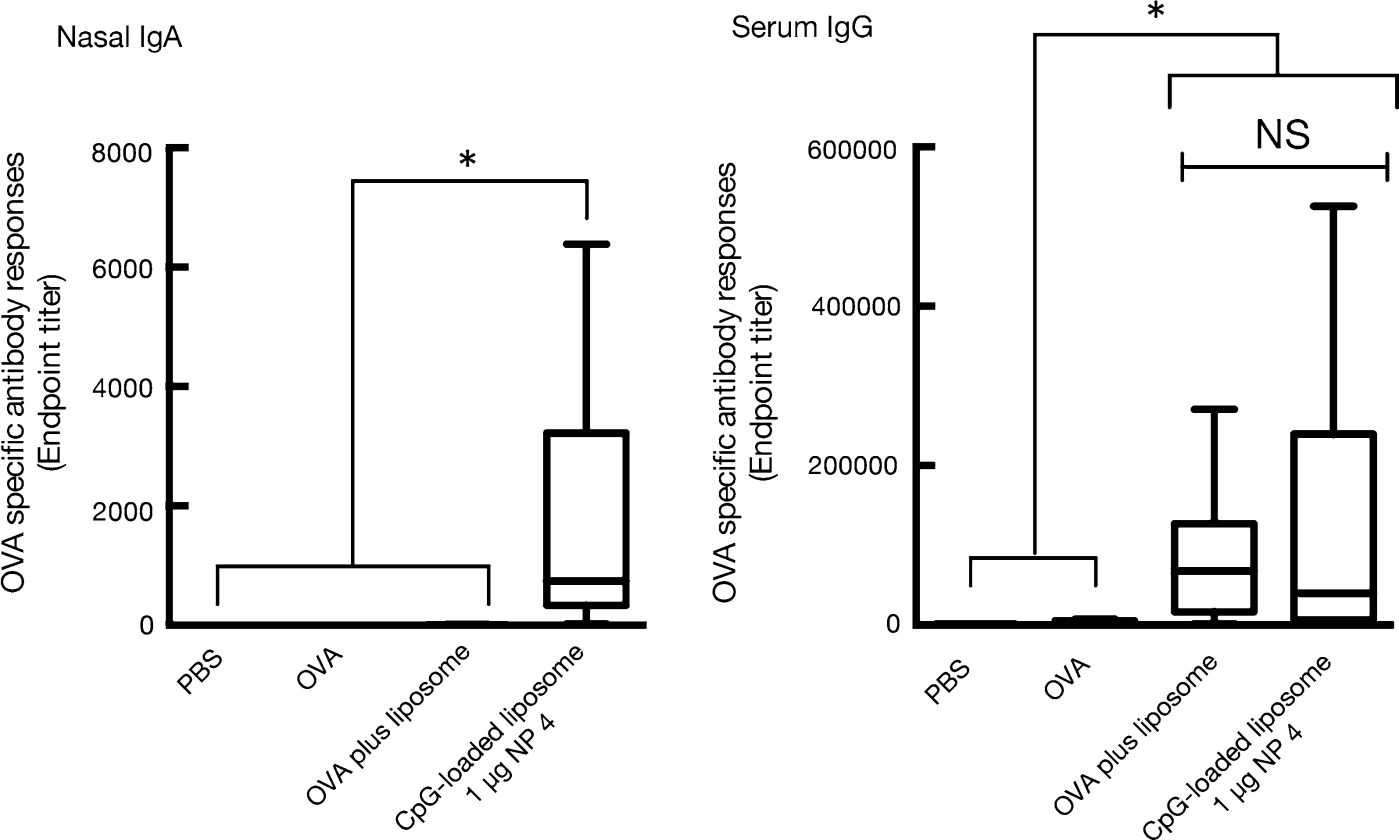
Mucosal adjuvant activity of CpG ODN-loaded DOTAP/DC-chol liposomes assessed by the induction of OVA-specific nasal IgA and serum IgG in BALB/c mice. BALB/c female mice were immunized intranasally with PBS, OVA (5 µg/mouse) alone, OVA (5 µg/mouse) plus DOTAP/DC-chol liposomes, OVA (5 µg/mouse) plus CpG ODN (1 µg/mouse), or OVA (5 µg/mouse) plus CpG ODN-loaded DOTAP/DC-chol liposomes on days 0 and 7. Serum and nasal washes were collected on day 14. The anti-OVA IgA level in nasal washes and anti-OVA IgG, IgG1, and IgG2a levels in serum were detected by ELISA assay as described in the “Methods” section. The data were obtained from three independent experiments. The box-plot shows the median value with the 25th–75th percentiles and the *error bars* indicate the 5th–95th percentiles. The *symbols in box plots* represent individual mice (PBS, n = 10; OVA, n = 10; OVA plus liposome, n = 10; OVA plus CpG-loaded liposome, n = 12). Significance was assessed using the Kruskal–Wallis with Dunn’s post–hoc test: *p < 0.05, *NS* not significant

To evaluate the impact of CpG ODN attached to the cationic liposomes, DOTAP/DC-chol liposomes, we next examined whether the augmentation of the mucosal adjuvant activities of CpG ODN-loaded DOTAP/DC-chol liposomes could be observed compared with those of CpG ODN alone. Namely, we explored the mucosal adjuvant activities of CpG ODN-loaded DOTAP/DC-chol liposomes at the N/P ratio of 4. In case of IgA induction in the nasal area, 1 µg of CpG ODN-loaded DOTAP/DC-chol liposomes synergistically induced IgA production (endpoint titer with median value; 738.8). These values are more than 10 times higher than those induced by free CpG ODN. Interestingly, the DOTAP/DC-chol liposomes did not induce any antigen-specific IgA responses in the nasal area at the dose used (8.52 nmol/mouse), which is similar to our previous study [29]. In contrast to IgA production, the antigen-specific IgG responses did not change even when CpG ODN was loaded onto the liposomes as DOTAP/DC-chol liposome alone strongly induces antigen-specific IgG production (Fig. 4).

**Fig. 5:**
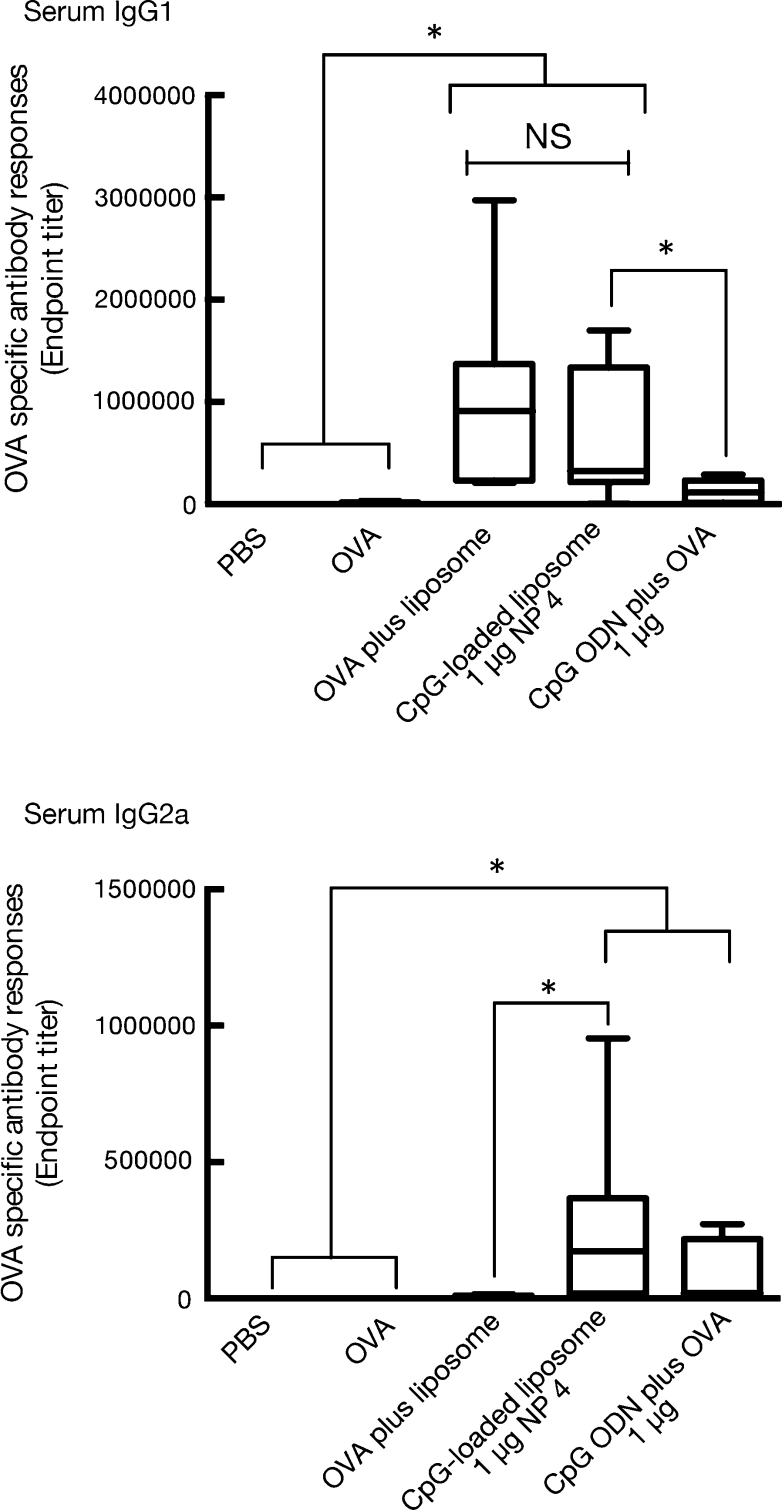
Serum IgG subclasses induced by intranasal immunization of OVA plus CpG ODN-loaded DOTAP/DC-chol liposomes in BALB/c mice. BALB/c female mice were immunized intranasally with PBS, OVA (5 µg/mouse) alone, OVA (5 µg/mouse) plus DOTAP/DC-chol liposomes, or OVA (5 µg/mouse) plus CpG ODN-loaded DOTAP/DC-chol liposomes on days 0 and 7. Serum samples were collected on day 14. The anti-OVA IgG1 and IgG2a levels in serum were detected by ELISA assay as described in the “Methods” section. The data were obtained from three independent experiments. The *box-plot* shows the median value with the 25th–75th percentiles and the *error bars* indicate the 5th–95th percentiles. The *symbols in box plots* represent individual mice (PBS, n = 10; OVA, n = 10; OVA plus liposome, n = 10; OVA plus CpG-loaded liposome, n = 12; OVA plus CpG ODN, n = 8). Significance was assessed using the Kruskal–Wallis with Dunn’s post hoc test: *p < 0.05, *NS* not significant

Since murine serum IgG subclasses have been used to assess the type of immune responses [36, 37], we investigated the production of serum IgG1 and IgG2a after intranasal immunization with OVA and CpG ODN-loaded DOTAP/DC-chol liposomes. Our results (Fig. 5) reveal that serum IgG1 responses remained unchanged for both DOTAP/DC-chol liposomes and CpG ODN-loaded DOTAP/DC-chol liposomes, indicating that serum IgG1 responses (referred to as Th2 response) largely relied on the DOTAP/DC-chol liposomes, which act as a Th2-type immune adjuvant by inducing a Th2 cytokine, interleukin-4 (IL-4) [29]. For serum IgG2a responses, the CpG ODN-loaded DOTAP/DC-chol liposomes synergistically induced the production of serum IgG2a (indicated as a Th1 response), which largely depended on the effect of CpG ODN. CpG ODNs are known to strongly induce Th1-associated cytokines, such as interferon-γ (IFN-γ) and interleukin-12 (IL-12) from natural killer cells (NKs) and macrophages, respectively [14]. These cytokines mainly promote cellular immunity also referred to as Th1 response. In this study, the samples for the determination of antibody production were collected on day 14. Generally, on day 14, post initial immunization, antibody responses do not reach maximum levels because of exposure only to primary antigen. In such cases, maximum antibody production would normally take about 30 days post exposure to the antigen. Furthermore, the peak of hypermutated high-affinity IgG antibodies generated via the germinal center reaction is reached 4–6 weeks post exposure. In this study, mice were administered the antigen with mucosal adjuvant twice: the first dose followed by a booster dose. It is well demonstrated that booster exposure to the antigen, in secondary immune responses, reactivates immune memory and results in a rapid increase of antibody titers (typically reaching maximum antibody response within 7 days) [38, 39]. Therefore, the immunization schedule used in this study was ideal for the comparison of the mucosal adjuvant activity between different sets of adjuvants. The results obtained here indicate that the combination of CpG ODN and DOTAP/DC-chol liposomes exert balanced Th1/Th2 immune responses, suggesting that CpG ODN-loaded DOTAP/DC-chol liposomes can act as a nanoparticle-based mucosal adjuvant system. The detailed mechanism(s) responsible for the effective induction of antigen-specific mucosal immune responses by CpG ODN-loaded DOTAP/DC-chol liposomes remain ambiguous. It is possibly caused by a more efficient delivery of both CpG ODN and OVA to the APCs by DOTAP/DC-chol liposomes. In an earlier study, we have already reported that the DOTAP/DC-chol liposomes promote antigen-uptake by APCs in vivo [29]. Additionally, recent reports have revealed that the combination of multiple adjuvants synergistically stimulated APCs [40], therefore we hypothesized that DOTAP/DC-chol liposomes can induce endogenous immunostimulatory molecules in the nasal area, such as adenosine triphosphate (ATP), high mobility group box–1 (HMGB1), and host double stranded DNA that are considered as an damage-associated molecular pattern (DAMP) to trigger an innate immune response. Therefore, we are now investigating whether the cationic liposomes exhibit host genomic DNA in the nasal area when administrated intranasally.

Taken together, CpG ODN-loaded DOTAP/DC-chol liposomes demonstrated higher mucosal adjuvant activity than free CpG ODN and induced of balanced Th1/Th2 reactions.

## Conclusions

In the present study, we showed that intranasal immunization of a model antigen, OVA, with class B CpG ODN-loaded DOTAP/DC-chol liposomes synergistically induced both antigen-specific mucosal IgA and systemic IgG responses in mice, clearly demonstrating that the attachment of CpG ODN onto the cationic liposomes remarkably enhanced mucosal adjuvant effects. These aspects allow us to reduce the dose of CpG ODN in nasal vaccines. Vaccine system using liposomes have advantages because liposomes are biodegradable within host and are essentially non-antigenic. Moreover, liposomal vehicle is easily able to deliver to the intended tissues and/or cells by modifying their liposomal surface with proteins or carbohydrates. These characteristics are believed to be superior to that of the existing mucosal adjuvants. However, the underlying mechanisms involved in the effect of the mucosal adjuvant are not yet fully understood, the potent mucosal adjuvant activity of CpG ODN-loaded DOTAP/DC-chol liposomes might help in developing an appropriate strategy for mucosal vaccine therapy to prevent and treat infectious diseases.

## Abbreviations

APCs: antigen-presenting cells
CpG ODN: oligodeoxynucleotides containing immunostimulatory CpG motifs
DAMPs: damage-associated molecular patterns
DC-chol: 3β-[*N*-(*N*′,*N*′-dimethylaminoethane)-carbamoyl]
DOTAP: 1,2-dioleoyl-3-trimethylammonium-propane
IFN-γ: interferon-γ
IL-12: interleukin-12
IL-4: interleukin-4
ODNs: oligodeoxynucleotides
OVA: ovalbumin from egg white
PBS: phosphate-buffered saline
PO: phosphodiester
PS: phosphorothioate
TMB: tetramethylbenzidine
TLR9: toll-like receptor 9
